# Trends in publications regarding evidence-practice gaps: A literature review

**DOI:** 10.1186/1748-5908-5-11

**Published:** 2010-02-03

**Authors:** Ann E Evensen, Rob Sanson-Fisher, Catherine D'Este, Michael Fitzgerald

**Affiliations:** 1 Department of Family Medicine, University of Wisconsin School of Medicine and Public Health, 100 North Nine Mound Road, Verona, Wisconsin, USA; 2 Faculty of Health, School of Medicine and Public Health, University of Newcastle, 345 David Maddison Building, Watt and King Streets, Newcastle, Australia; 3 Centre for Clinical Epidemiology and Biostatistics, School of Medicine and Public Health, Faculty of Health, University of Newcastle, University Drive, Callaghan, Australia

## Abstract

**Background:**

Well-designed trials of strategies to improve adherence to clinical practice guidelines are needed to close persistent evidence-practice gaps. We studied how the number of these trials is changing with time, and to what extent physicians are participating in such trials.

**Methods:**

This is a literature-based study of trends in evidence-practice gap publications over 10 years and participation of clinicians in intervention trials to narrow evidence-practice gaps. We chose nine evidence-based guidelines and identified relevant publications in the PubMed database from January 1998 to December 2007. We coded these publications by study type (intervention versus non-intervention studies). We further subdivided intervention studies into those for clinicians and those for patients. Data were analyzed to determine if observed trends were statistically significant.

**Results:**

We identified 1,151 publications that discussed evidence-practice gaps in nine topic areas. There were 169 intervention studies that were designed to improve adherence to well-established clinical guidelines, averaging 1.9 studies per year per topic area. Twenty-eight publications (34%; 95% CI: 24% - 45%) reported interventions intended for clinicians or health systems that met Effective Practice and Organization of Care (EPOC) criteria for adequate design. The median consent rate of physicians asked to participate in these well-designed studies was 60% (95% CI, 25% to 69%).

**Conclusions:**

We evaluated research publications for nine evidence-practice gaps, and identified small numbers of well-designed intervention trials and low rates of physician participation in these trials.

## Background

Many clinical guidelines have not been fully implemented in clinical practice, despite widespread acceptance of evidence-based recommendations by the medical community [1-21]. Closing these 'evidence-practice gaps' would result in significant improvements in public health. This outcome is desirable, but requires removal of barriers at the level of the patient, physician, medical organization, and socioeconomic or political community [22,23].

Researchers and clinicians who identify specific barriers to guideline adoption and then design interventions to purposefully overcome them are most likely to affect change [24]. This process requires well-designed trials to identify the most successful strategies for change. This type of research is called 'knowledge translation' or T2, 'the translation of results from clinical studies into everyday clinical practice and health decision making' [25]. Funding for T2 research lags significantly behind that for technological innovations, despite estimates that health outcomes are more likely to improve with universal adoption of already proven guidelines [26,27].

Despite the absence of proven strategies for guideline implementation, physicians are expected to successfully adopt guidelines into their practices. Physicians are held accountable for evidence-practice gaps when their practices are measured by internal quality reviews, insurance companies and government entities, (*e.g*., 'pay for performance') [28].

We hypothesized that the demand on physicians and health systems for improved patient outcomes would create demand for evidence-based methods for incorporating guidelines into clinical practice. We expected that the number of methodologically rigorous trials examining the differential effectiveness of strategies to change the behavior of clinicians or function of health care systems would increase over time. We also expected that there would be high levels (>75%) of clinician participation in such trials.

## Methods

### Literature search

We conducted a literature search to identify relevant publications. We examined English language studies regarding nine guidelines (Table 1) from January 1998 to December 2007 by performing a computer-based literature search of the PubMed data base with the following search terms: (clinical performance OR attitude OR knowledge OR evidence practice gap OR practice guidelines as topic* [mh] OR guideline adherence [mh] OR clinical practice guideline* OR guideline* [title] OR recommendation* OR adherence OR best practice* OR implementation OR know to do gap OR knowledge translation) AND (('1998/01/01' [PDat]: '2007/12/31' [PDat]) AND (English [lang])) AND (topic area term). The first and second authors selected the nine practice guidelines for analysis. Guidelines met all of the following criteria: each guideline was broadly applicable to the practice of family medicine; each guideline was supported by well-designed clinical trials; and we could identify a persistent evidence-practice gap for each guideline [1-10,16-21]. A persistent gap was determined to be present if demographic studies quantified a gap prior to January 1998 and after December 2007 and are referenced in Table 1. Analysis was limited to the practice recommendations listed in Table 1. Other practice recommendations included in the referenced guidelines were not included in this analysis.

**Table 1:** Medical guidelines selected for analysis

Topic	Guideline
ACE inhibitors	ACE inhibitors are the agent of choice in treatment of hypertension in diabetes mellitus.

Beta-blockers	Beta blockers should be prescribed to patients who have experienced a myocardial infarction.

Asthma	Inhaled anti-inflammatory agents should be used in patients with persistent asthma.

Atrial fibrillation	Patients with atrial fibrillation should be anticoagulated with coumadin.

Pain in cancer patients	Pain should be treated aggressively in terminal cancer patients.

Antibiotics for URTI	Antibiotics should not be used to treat viral upper respiratory tract infections.

Smoking in pregnancy	Pregnant women should be counselled to quit smoking.

Cervical cancer screening	Adult women should have regular cervical cancer screening.

Breast cancer screening	Adult women should have regular mammograms.

References [1-21]

### Article classification

We initially divided articles in each of nine topics into two categories: intervention studies and non-intervention studies or publications. Intervention studies were defined as those that evaluated strategies to close the evidence-practice gap by changing patient or clinician attitudes, clinical behavior, and/or knowledge. If a publication incorporated intervention and non-intervention elements, it was included as an intervention study.

We further subdivided intervention studies based on the target of the intervention (patient or clinician). 'Patient' studies were defined as those that had the intervention applied to patients or family caregivers of patients. For example, a trial that compared rates of mammography in women randomized into two groups (advising by lay health advisors versus no intervention) would be a 'patient' study. 'Clinician' studies were defined as those that had interventions applied to clinicians or the health system. For example, a trial that compared antibiotic prescribing practices of physicians randomized into two groups (guideline dissemination by mail versus discussion of guidelines in a small group of physicians) would be a 'clinician' study.

We classified a study that evaluated interventions targeting both patients and clinicians as a 'clinician' study. We then classified 'clinician' intervention studies using standard Effective Practice And Organization Of Care (EPOC) criteria for research design into 'well-designed studies' (EPOC criteria 1.1-1.2, inclusive, describing randomized controlled trials (RCTs), controlled clinical trials, controlled before and after studies with adjustment for confounders, and interrupted time series) [29] and 'other studies' (any studies that did not meet EPOC criteria for adequate research design).

We subdivided non-intervention publications based on primary content (editorial, descriptive study, or treatment guideline). 'Editorial' publications were defined as non-data-based studies offering commentary on a facet of the evidence-practice gap. 'Descriptive studies' were data-based examinations of the evidence-practice gap, such as studies of epidemiology or sociodemographic factors, but did not evaluate any intervention strategy. 'Treatment guidelines' were defined as publications that described current treatment recommendations or clinical guidelines and did not report original research. Studies that examined the efficacy of treatment recommendations were excluded. Ten percent of the abstracts were randomly selected and type of study independently re-coded to provide an estimate of inter-rater reliability.

### Statistical Methods

We investigated: whether the total number of evidence-practice gap publications that evaluated intervention strategies designed to improve clinician adherence to best practices increased over time; whether the proportion of these publications that were intervention studies increased over time; the proportion of these interventions that would be adequate as defined by EPOC criteria for experimental design [29]; and clinician participation in well-designed intervention trials.

The number of publications for each topic and type of study are presented. Given the small number of publications, studies were collapsed across topic areas and analysis undertaken on the pooled studies for the remaining analyses.

We undertook linear regression analysis of the number of evidence-practice gap articles versus time. A regression coefficient that was statistically significantly different from zero indicated an increase in the number of publications over time. We used the Cochran-Armitage Trend Test to determine whether the proportion of selected evidence-practice gap publications that were classified as intervention studies increased over time.

The percentage of clinician-focused intervention studies that used an adequate design (by EPOC criteria) was calculated with a 95% confidence interval (CI). If the lower limit of the confidence interval is greater than 75%, then we can conclude that the proportion of intervention studies that are RCTs is greater than a hypothesized value of 75%. Seventy-five percent was pre-specified in this analysis by consensus of the authors that this figure represented a clear majority of studies.

The median clinician consent rate for all studies targeting clinician adherence to best practice was calculated with a 95% confidence interval and compared to a hypothesized value of 75%.

### Inter-rater reliability

We calculated the Kappa statistic to assess agreement between the two raters on type of study.

No approval was required by a human-subjects review board.

## Results

For the nine medical guidelines, we identified 1,151 relevant publications from January 1998 to December 2007.

### Total number of evidence-practice gap studies over time

The number of studies on the evidence-practice gap in the defined areas varied from 85 in 1998 to a high of 145 in 2003 (Tables 2 and 3). The slope of the simple linear regression model for total number of evidence-practice gap studies versus year was 2.10 (95% CI, -2.46 to 6.66), indicating no statistically significant increase over time.

**Table 2:** Number of intervention studies by year and topic

Year	1998	1999	2000	2001	2002	2003	2004	2005	2006	2007	Total
**Topic**											

Mammograms	7	8	3	2	9	5	5	11	4	8	62

Beta-blockers	2	4	0	3	1	1	3	0	3	2	19

Atrial fibrillation	0	3	0	0	1	0	1	1	2	4	12

ACE inhibitors	0	0	0	0	0	1	1	0	1	0	3

Pain in cancer patients	0	1	0	0	0	0	1	0	0	0	2

Cervical cancer screening	1	0	0	0	0	0	0	0	1	1	3

Smoking in pregnancy	1	0	4	3	4	3	2	0	3	2	22

Antibiotics for URTI	0	3	4	3	5	3	4	6	6	8	42

Asthma	0	1	0	0	1	0	1	0	1	0	4

**TOTAL**	**11**	**20**	**11**	**11**	**21**	**13**	**18**	**18**	**21**	**25**	**169**

**Table 3:** Proportion of pooled intervention studies by year

Year	Total evidence-practice gap studies	Evidence-practice gap intervention studies (percent of total studies)
1998	85	11 (13%)
1999	114	20 (18%)
2000	102	11 (11%)
2001	129	11 (8.5%)
2002	113	21 (19%)
2003	145	13 (9.0%)
2004	123	18 (15%)
2005	117	18 (15%)
2006	93	21 (23%)
2007	130	25 (19%)
Total	1151	169 (15%)

### Proportion of intervention trials compared to total evidence-practice gap studies

We found 169 intervention studies (15%) and 982 non-intervention studies (85%) (Tables 2 and 3). The percentage of all evidence-practice gap publications that involved intervention studies ranged from a minimum of 8.5% in 2001 to a maximum of 23% in 2006, a trend over time that was marginally non-significant (Cochran Armitage Trend Test Z = 1.9514, p = 0.0510).

### Proportion of intervention studies that were well-designed

Of the 169 intervention trials, 87 (51%) were intended for patients and 82 (49%) were intended for clinicians. Of the 82 interventions intended for clinicians, 28 (34%; 95% CI, 24% to 45%) met the EPOC criteria for well-designed studies. Thus, the majority of intervention studies for clinicians do not meet EPOC criteria for well-designed studies. Of the studies that met EPOC criteria, there were 14 RCTs, two controlled clinical trials, five controlled before-and-after studies, and seven interrupted time designs. The most common reason for failure to meet EPOC criteria for good design was the inclusion of only one data point measurement of adherence to best practices before and after introduction of a guideline (28 of 54 studies). The remaining studies that failed to meet EPOC criteria were surveys, interviews, pilot projects, and observational studies.

### Clinician consent rate in well-designed intervention studies

Only 11 of the 28 clinician-focused evidence-practice gap intervention studies meeting the EPOC criteria were included in this analysis, as 13 studies did not mention consent rates and four studies listed consent at the level of the physician practice or peer group rather than the individual physician. The median consent rate for well-designed studies targeting clinician adherence to best practice was 60% (95% CI, 25-69%), which was not greater than the hypothesized value of 75%.

### Inter-rater reliability

In all, 109 publications were independently re-classified by type of study resulting in a Kappa of 0.85 (95% CI, 0.77 to 0.93).

## Discussion

We examined publications of the last ten years related to the persistent evidence-practice gap in nine medical topic areas. We chose these topic areas because each guideline is supported by well-designed clinical trials and has been accepted by the medical community for a minimum of ten years [1-4,7-10]. Despite widespread support for routine use of these guidelines to decrease morbidity, mortality, and/or costs, an ongoing evidence-practice gap is identified for each guideline [5,6,11-21].

If the evidence-practice gaps were closing, it would be reasonable that further study of interventions or how to adopt them would not be needed. Because we document that gaps in all nine clinical topic areas are persisting, we expect that meaningful research would be ongoing. This research should include trials of strategies to affect change [24]. In contrast, we document with this study that over time the number of articles about the nine defined evidence-practice gaps did not significantly increase (2.10, 95% CI, -2.46 to 6.66). Our analysis demonstrated a marginally non-significant increase over time in the proportion of evidence-practice gap studies that were intervention studies, indicating that there may be some evidence of an increasing trend. However, the total number of intervention studies remained surprisingly low (an average of 1.9 intervention trials per year per topic area). The majority (53%) of publications over a ten-year period fell instead into the 'descriptive' category (see Methods for classification parameters). Although data-based, descriptive studies can only define or highlight problems rather than test solutions.

Reasons for the limited number of interventions were not identified by this study, but other reviews suggest ethical concerns, funding restrictions, and degree of difficulty in completing controlled trials compared with descriptive studies [22,23,26,27].

Moving beyond observational studies, pre-post evaluations, and pilot studies to well-controlled research is necessary to obtain valid and generalisable results. However, we found few of these high-quality studies. Only 28 studies in nine subject areas over a ten-year period were well-designed studies evaluating strategies that clinicians or health systems could use to improve adherence to best practices. These 28 studies represent 16% of the total intervention studies, and only 2.4% of all of the evidence-practice gap publications in the nine topic areas.

The research patterns we describe above are discouraging, and it would be easy as a practicing physician to lay blame on the researchers or funding agencies designing and/or choosing which grants to support. It is also reasonable that a practicing physician may decline to participate in sloppy or frivolous research. However, this study documents low participation rates in clinician intervention studies that met EPOC criteria for adequate design. The median physician consent rate in well-controlled trials was 60% (95% CI, 25 to 69%).

Physicians may not participate in intervention trials for many reasons, including financial or time constraints, failure to be invited to participate, lack of interest, and disagreement about the medical merits of the intervention or research goal. A clinician may also refuse due to more psychologically complex issues: trial participation requires an acknowledgement that evidence-practice gaps exist and a willingness to let others dictate one's behavior. Physicians may also lack confidence that they are suitable agents of change for these guidelines. However, the medical community expects patients to readily participate in clinical trials so that valid and generalisable results are obtained. Physicians should be held to the same standard of participation.

Limiting the search to the PubMed database may have resulted in missing some relevant publications, but it is likely that a high-quality study would be published in a peer-reviewed journal catalogued in PubMed. The choice of medical guidelines may also affect the search results, but a similar pattern of limited high-quality interventions was seen in every guideline examined.

## Summary

Evidence-practice gaps for nine well-established medical guidelines have persisted for the past ten years. Publications regarding these gaps are consistently descriptive in nature or simply restate treatment recommendations, with few rigorous trials of methods for closing the evidence-practice gap. The scarcity of high-quality intervention trials and low physician participation in these trials decrease the likelihood of closing the evidence-practice gap. This research pattern is insufficient to create successful strategies for implementing best practices. Instead, physicians are left without reliable means to improve their patients' health or means to meet the demand for improved health outcomes from employers and insurers. A new research pattern of evaluating strategies for changing clinical behavior and the functioning of health care systems is needed. Individual clinicians should contribute to translational research by readily agreeing to participate in these trials.

## Competing interests

The authors declare that they have no competing interests.

## Authors' contributions

RSF and AE conceived and designed the study. AE collected the data. All authors analyzed and interpreted the data. All authors drafted and revised the manuscript, and approved the final version.
